# Prognostic significance of PD‐L2 expression in patients with oral squamous cell carcinoma—A comparison to the PD‐L1 expression profile

**DOI:** 10.1002/cam4.1929

**Published:** 2019-01-18

**Authors:** Manuel Weber, Falk Wehrhan, Christoph Baran, Abbas Agaimy, Maike Büttner‐Herold, Marco Kesting, Jutta Ries

**Affiliations:** ^1^ Department of Oral and Maxillofacial Surgery Friedrich‐Alexander University Erlangen‐Nürnberg (FAU) Erlangen Germany; ^2^ Institute of Pathology Friedrich‐Alexander University Erlangen‐Nürnberg (FAU) Erlangen Germany; ^3^ Institute of Pathology, Department of Nephropathology Friedrich‐Alexander University Erlangen‐Nürnberg (FAU) Erlangen Germany

**Keywords:** oral cancer, OSCC, PD‐L1, PD‐L2, peripheral blood, RT‐qPCR

## Abstract

**Background:**

Despite the observed association of increased PD‐L1 expression in peripheral blood of oral squamous cell carcinoma (OSCC) patients with histomorphologic parameters, the role of the PD1 ligands—PD‐L1 and PD‐L2—is insufficiently understood. Aim of the study was to investigate whether the alterations of PD‐L1 and PD‐L2 expression in blood are associated with survival and could serve as immune monitoring parameter. Moreover, it should be analyzed if PD‐L2 is differentially expressed in tissue and blood samples of OSCC patients compared to healthy controls and if there is an association of PD‐L2 expression with histomorphologic and prognostic tumor parameters.

**Methods:**

PD‐L2 mRNA expression was analyzed in tumors and healthy oral mucosa specimens and in corresponding peripheral blood samples of 48 OSCC patients and 26 healthy controls using RT‐qPCR. A cutoff point (COP) was determined and a chi‐square test (*χ* ^2^ test) was carried out. Survival analysis of PD‐L2 and previously reported PD‐L1 expression data was performed using Kaplan‐Meier analysis (Log‐rank test).

**Results:**

PD‐L2 expression in tissue samples was significantly (*P* < 0.001) higher in OSCC patients compared to healthy controls. A significant association of PD‐L2 expression above the COP (positive) with malignancy was ascertained (*P* < 0.001). A significant (*P* = 0.01) association of previously reported PD‐L1 expression rates in peripheral blood with survival could be shown.

**Conclusion:**

Peripheral blood PD‐L1 expression might be a prognostic marker for OSCC patients and a possible parameter to monitor immune dysfunction in malign diseases. In the peripheral blood, PD‐L1 might be more relevant for immune tolerance than PD‐L2. Local PD‐L2 expression in tissue samples might be useful as a diagnostic parameter for malignancy and could contribute to the immunosuppressive local microenvironment in OSCC.

## 1 INTRODUCTION

Surgical tumor resection and radiotherapy have proven to be the most effective treatment options for oral squamous cell carcinomas (OSCC). However, advanced and relapsed OSCC after exhaustion of the conventional therapies lacks efficient therapeutic options.^1^ Since the introduction of inhibitors of the PD1 immune checkpoint pathway a relevant number of these patients show long‐time survival.^2^ For this reason, head and neck tumors such as OSCC are a main target of the current clinical studies in immune‐oncology.^3^

Despite these encouraging results, it needs to be stated that about 80% of advanced OSCC patients do not respond to anti‐PD1 treatment.^4^ Although a high expression of the PD1 ligand PD‐L1 was associated with superior response to anti‐PD1 therapies in drug approval studies of Nivolumab and Pembrolizumab,^2^, ^5^ a relevant proportion of immunohistochemically PD‐L1 negative patients does also respond to anti‐PD1 treatment. The current use of immune checkpoint inhibitors in OSCC was enabled by translating results from clinical studies in other tumor entities. Moreover, a large discrepancy exists between successful clinical targeting and incomplete understanding of the biologic role of the PD1 pathway. Compared to the main ligand PD‐L1 which is increasingly studied, the physiologic and pathophysiologic relevance of PD‐L2 is poorly understood.^6^

Hence, there is no reliable predictive marker available to select patients who will benefit from the checkpoint blocking immunotherapy,^3^, ^4^ and currently, no recommendation for testing PD‐L1 expression in oral cancer prior to treatment with checkpoint inhibitors is given.^3^ One possible explanation for the efficiency of PD1‐blocking in PD‐L1 negative tumors might be the inhibition of PD‐L1/PD1 interaction in circulating immune cells.^7^ Another explanation could be the activation of PD1 by other ligands such as PD‐L2.^8^ However, the expression profile of PD‐L2 in OSCC patients is poorly investigated and needs further research to determine the impact in immunomodulation in OSCC.

PD‐L2 was first described in 2001 as second ligand for the PD1 receptor.^6^ PD‐L1 and PD‐L2 share about 40% of their amino acid sequence.^4^ The distribution of PD‐L1 and PD‐L2 in different cell types is differentially regulated. PD‐L1 is physiologically expressed by immune cells as well as in lung tissue, vascular endothelium, mesenchymal stem cells, astrocytes, and keratinocytes.^9^ PD‐L2 expression is more restricted to antigen‐presenting cells and does not show physiologically relevant expression in activated T cells or keratinocytes.^4^, ^9^ PD/PD‐L signaling is likely not monodirectional from antigen‐presenting cells or tumor cells to T cells. PD1 and its ligands can be expressed on T cells, B cells, and antigen‐presenting cells—indicating reverse and possibly bidirectional signaling. Soluble PD1 was found to inhibit dendritic cell activation and to increase the expression of immunosuppressive mediators such as IDO and IL‐10.^9^ These data outline the complexity and point out unsolved questions in PD/PD‐L signaling.

PD‐L2 is gaining importance in the complex network of cancer immune tolerance and immune therapy response. However, in contrast to PD‐L1, expression of PD‐L2 in human tumors is far less investigated. Recently, a significantly increased survival of PD‐L2‐positive head and neck squamous cell carcinoma (HNSCC) patients treated with the PD1‐inhibitor pembrolizumab was shown.^8^ In hepatocellular carcinoma and in gastric adenocarcinoma, both increased PD‐L1 and PD‐L2 expression were associated with inferior prognosis,^10^, ^11^ outlining the role of PD‐L2 as immunosuppressive and cancer‐promoting signaling molecule. In salivary gland tumors, increased PD‐L2 expression was shown to be associated with disease relapse.^12^ In Hodgkin lymphoma, PD‐L2 was expressed in fewer cases than PD‐L1. However, it was expressed in some PD‐L1 negative cases.^13^ In OSCC, systematic assessment of PD‐L2 expression is missing.^14^

In a previous study, we could show that PD‐L1 expression in OSCC tissue is significantly upregulated compared to healthy oral mucosal specimens.^7^ Additionally, patients with lymph node metastases (N+) showed significantly increased PD‐L1 expression in peripheral blood compared to N0 patients.^7^ These results underline that besides local expression in tumor tissue, the systemic immune cell‐based expression of immune checkpoints should be considered. A recent flow cytometric analysis in advanced lung cancer also supports the concept of systemic immune checkpoint expression as an association of increased numbers of PD‐L1 and PD‐L2‐positive T cells in peripheral blood with inferior survival was shown.^15^ This underlines the importance of considering PD‐L2 expression additional to PD‐L1 to better understand the tumorbiologic significance of PD1‐signaling.

In the 1980, immune monitoring parameters in OSCC patients were investigated for the first time.^16^, ^17^, ^18^ However, the nonspecific systemic immune reactivity parameters available at that time led to contradictory conclusions^16^, ^18^ and could not be established as prognostic parameters.^18^ In contrast to these previous approaches with unspecific parameters, today the expression of therapeutically highly relevant immune checkpoints can be measured specifically.

This study aimed to elucidate the value of PD‐L2 as immune monitoring parameter in OSCC compared to PD‐L1. Therefore, PD‐L2 expression in tissue specimens and peripheral blood samples of OSCC patients compared to controls was assessed using RT‐qPCR. It should be tested if PD‐L2 expression in OSCC tissue is different from healthy oral mucosa. Additionally, it was analyzed whether PD‐L2 expression in peripheral blood samples of cancer patients is altered in comparison to peripheral blood of healthy control persons. Alterations in PD‐L2 expression should be compared to PD‐L1. Moreover, it was addressed whether altered PD‐L1 and PD‐L2 expression in peripheral blood influences the survival of OSCC patients.

## 2 MATERIAL AND METHODS

### 2.1 Patients and sample collection

The study was performed in accordance with the Declaration of Helsinki and approved by the Ethics Committee of the University of Erlangen‐Nuremberg, Erlangen, Germany (approval number: 3962). Patients’ written informed consent was obtained.

Tissue specimens and peripheral blood samples were collected from 48 OSCC patients (group patients) and 26 healthy volunteers (group controls). Patients were included in this study at first diagnosis of OSCC. All samples were taken before any treatment. Demographic characteristics including age and gender of all study participants are shown in Table 1. Healthy volunteers were selected based on the absence of inflammation and malignant disease. Tissue samples of healthy volunteers were taken during dentoalveolar surgery after informed consent.

**Table 1:** Description of the patient collective; total number of cases: 74

	Patents		Controls	
	n	% of cases	n	% of cases
Number of cases	48		26	
Gender				
Male	34	70.8	17	65.4
Female	14	29.2	9	34.6
Mean age	62.7 y (SD 12.5)		54.4 y (SD 22.7)	
Age range	35‐93 y		15‐88 y	
T‐status				
T1‐T2	29	60.4		
T3‐T4	18	37.5		
Unknown	1	2.1		
N‐status				
N0	25	52.1		
N+	22	45.8		
Unknown	1	2.1		
L‐status				
L0	37	77.1		
L1	10	20.8		
Unknown	1	2.1		
Pn‐status				
Pn0	29	60.4		
Pn1	18	37.5		
Unknown	1	2.1		
Grading				
G1	7	14.6		
G2	23	47.9		
G3	18	37.5		
Clinical stage				
Early	17	35.4		
Late	30	62.5		
Unknown	1	2.1		

Grading, histologic tumor grading; L‐status, lymph vessel invasion; n, number of cases; N‐status, lymph node metastases; Pn‐status, perineural invasion; SD, standard deviation; T‐status, tumor size.

Demographic characteristics of OSCC patients (group patients) and healthy volunteers (group controls) for PD‐L2 analysis. For the OSCC patients group, staging parameters (T‐, N‐, L‐, Pn‐status, grading, clinical UICC stage) are shown.

The diagnosis of OSCC was assigned through routine histopathological examination. Grading (G1‐G3; differentiation status), clinical UICC stage (I‐IV), and TNM classification of OSCC were documented according to the guidelines of World Health Organization classification of tumors of the head and neck and the International Union Against Cancer.^19^, ^20^ Clinical stages were grouped as early (stage I and II) and late (stage III and IV) stages. Additionally, lymph node status was gathered as N0 and N+ to indicate the absence (N0) or presence (N+) of metastases, respectively. Additionally, subgroups were categorized based on tumor size for small (T1/T2) and large (T3/T4) malignancies. Moreover, survival data of the first 3 years after diagnosis were obtained from the “Tumorzentrum der Universität Erlangen‐Nürnberg.” Relevant clinical and histopathological parameters are summarized in Table 1.

### 2.2 Sampling of tumor specimens and whole peripheral blood

Tissue samples of healthy volunteers were obtained during minor surgery. All OSCC samples were obtained from surgical specimens removed during tumor excision. Each sample was divided into two pieces. One part was used for histological examination; the second part was immediately transferred into RNAlater^®^ (Qiagen, Hilden, Germany) and was fixed by incubation at 4°C for at least 24 hours. Afterward the specimens were stored at −80°C until mRNA isolation.

Additionally, two samples of 2.5 mL whole peripheral blood were collected in a PAXgene Blood RNA Tube (PreAnalytiX GmbH, Hombrechtikon, Switzerland) from healthy volunteers as well as from OSCC patients before surgery and tumor removal, respectively. The samples were incubated at room temperature for two hours and stored at −20°C for at least 24 hours. Long‐term storage up to RNA isolation was carried out at −80°C.

### 2.3 Isolation of mRNA and RT‐qPCR analysis

RNA was extracted from tissue and blood samples using the miRNeasy mini‐Kit (Qiagen) and the PAXgeneBlood miRNA Kit (PreAnalytiX GmbH), respectively. RNA samples were stored at −80°C until RT‐qPCR expression analysis.

Reverse transcription was done by the Transcriptor High‐Fidelity cDNA Synthesis Kit following the manufacturer`s recommendations (Roche, Mannheim, Germany). For amplification, the QuantiTect SYBR® Green PCR Kit (Qiagen) and gene‐specific primers for PD‐L2 and PD‐L1 were used (Table 2). Data acquisition and analysis were performed using the ABI Prism 7300 of Applied Biosystems (ThermoFisher Scientific Inc, Waltham, MA, USA). The normalization of the values of RT‐qPCR analyses was done by the ΔCT method using the house‐keeping gene GAPDH as internal control. The relative quantification of differences in gene expression between the two groups was based on the ∆∆CT method. The relative difference in expression rates (RQ) between the two groups was calculated by the formula 2^−ΔΔCT^.

**Table 2:** Real‐time qPCR primer

Primer	Sequence (5' to 3')	Primer (bp)	Amplicon (bp)	Annealing temperature (°C)	Accession
PD‐L2 in/s	ACAGTGCTATCTGAACCTGTGG	22	98	60	NM_025239.3
PD‐L2 in/as	CTGCAGGCCACCGAATTCTT	20	—	—	
PD‐L1_2^a^ s	AGACCACCACCACCAATTCC	20	173	60	NM_014143.3
PD‐L1_2^a^/as	TGGAGGATGTGCCAGAGGTA	20	—	—	
PD‐L1_4^b^/s	AGCTATGGTGGTGCCGACTA	20	152	60	NM_014143.3
PD‐L1_4^b^/as	CAGATGACTTCGGCCTTGGG	20	—	—	
GAPDH in/s	GACCCCTTCATTGACCTCAACTA	23	102	60	NM_002046.5
GAPDH in/as	GAATTTGCCATGGGTGGAAT	20	—	—	

bp (base pairs), s (sense), as (antisense).

The selected primers for RT‐qPCR mRNA expression analyses of PD‐L2 and PD‐L1.

a Amplification of transcript variant 1 and 2 simultaneously; amplicon named PD‐L1_2.

b amplification of transcript variant 1 and 4 simultaneously; amplicon named PD‐L1_4.

### 2.4 Statistical analysis of RT‐qPCR

The results of RT‐qPCR analyses were statistically evaluated applying the program IBM^®^ SPSS Statistics 22 (Chicago, IL, USA). All tests were based on the average of duplicate ΔCT values for each sample. First, the expression values were controlled for normal distribution by Shapiro‐Wilk test. Furthermore, the average values of all ΔCT within a group were calculated and used to determine the relative quantification (RQ) of the examined genes between the two groups by the ΔΔCT method. Twofold changes in mRNA expression rates (2 ≤ RQ ≤ 0.5) were defined as statistically relevant. Statistical relevance of the apparent expression between the two groups was analyzed by Mann‐Whitney *U* test. *P*‐values ≤0.05 were considered to indicate a statistically significant result. The ΔCT values were graphically plotted as Box‐Whisker plots which show the median, interquartile range, standard deviation, minimum, and maximum values of mRNA expression.

The discriminatory accuracy of the marker for distinguishing between the two groups was confirmed by receiver operator characteristic (ROC) curves. Additionally, the highest Youden index was determined. This value is associated with the threshold value, also named “cutoff point” (COP) for the biological marker. The COP defines the value of decreased or increased expression which is relevant for the discrimination between malignant and normal samples and allows the allocation of a particular sample to a certain group.^21^

Based on these COPs, the groups were divided into two subclasses which showed an expression rate over or under the COP. Afterward associations between altered mRNA expression and malignancy were calculated by the chi‐square test (*χ* ^2^ test).

### 2.5 Kaplan‐Meier analysis

In order to assess whether there is a statistically relevant correlation between overexpression of PD‐L2 in tissue and survival of the patients, a Kaplan‐Meier survival study was done. Moreover, survival analyses were performed based on the previously published PD‐L1 expression values in tissue and blood of the same patients.^7^ With regard to PD‐L1 expression, gene‐specific primers for the three biological active isoforms (transcript variants 1, 2, and 4) were used.^7^ Two splice variants (PD‐L1 variant 1 and 2 named amplicon PD‐L1_2 and PD‐L1 variant 1 and 4 named amplicon PD‐L1_4) were simultaneously amplified in each PCR. A COP distinguishing patients surviving 3 years from patients which deceased in this interval was determined. Kaplan‐Meier analysis was based on PD‐L1 expression levels above (positive) or below (negative) this COP. The significance was checked by the Log‐rank (Mantel‐Cox) test. *P* values <0.05 were considered as statistically significant.

## 3 RESULTS

### 3.1 Clinical and histomorphological parameters of the analyzed cases

Tissue specimens (OSCC and healthy oral mucosa) and whole blood samples of 48 OSCC patients (group patients) and 26 healthy volunteers (group controls) were investigated. Demographic characteristics of all participants and histomorphologic parameters of all OSCC patients are documented in Table 1. A total of 34 males and 14 females were included in the patients group. The control group consisted of 17 males and 9 females. Mean age was 62.7 years (SD ±12.5) in the patients group and 54.4 years (SD ±22.7) in the control group (Table 1). Both groups matched in age and gender (*P* > 0.05).

### 3.2 Comparison of PD‐L2 expression in tissue and blood between OSCC patients and healthy volunteers

OSCC patients (group patients) and healthy controls (group controls) were tested for significant differences in mRNA expression of PD‐L2. Higher ∆CT values indicate lower mRNA expression (Table 3).

**Table 3:** PD‐L2 expression in tissue and peripheral blood of healthy controls and OSCC patients

	n	Mean ∆CT	SD	*P*‐value	AUC	FC	COP	No. of cases	+	−	% pos. cases	*P*‐value *χ* ^2^ test	Sensitivity	Specificity	Pos. predictive value	Neg. predictive value
PD‐L2 tissue	61			<0.001	0.826	2.65	8.50	61	33	28		<0.001	77.8%	80.0%	0.848	0.714
Controls	25	9.16	0.93					25	5	20	20.0%					
Patients	36	7.76	1.33					36	28	8	77.8%					
PD‐L2 blood	72			0.797	nd	nd	nd	nd	nd	nd	nd	nd	nd	nd	nd	nd
Controls	24	10.95	1.38													
Patients	48	10.83	1.28													

−, positive cases in *χ* ^2^ test; +, positive cases in *χ* ^2^ test; AUC, area under the curve; COP, cutoff point; CT, cycle threshold; FC, fold change; grading, histologic tumor grading; L‐status, lymph vessel invasion; n, number of cases; nd, not determined; N‐status, lymph node metastases; Pn‐status, perineural invasion; SD, standard deviation; T‐status, tumor size.

Comparison of PD‐L2 mRNA expression between OSCC patients (group patients) and healthy volunteers (group controls). Expression of PD‐L2 in tissue (OSCC tumor tissue vs healthy oral mucosa of volunteers) and peripheral blood was analyzed. The mean ΔCT value (mean), standard deviation (SD), and the *P*‐value provided by the Mann‐Whitney U test are shown. Higher ΔCT values indicate lower PD‐L2 mRNA expression.

Regarding PD‐L2 expression in tissue, area under the curve (AUC), fold change (FC) and cutoff point (COP) values are given. Based on their ∆CT value related to the COP, the cases were determined as positive (malignant) and negative (healthy). The percentage of positive tested cases (% pos. cases) in the controls and patients group is presented. A statistical analysis was carried out by the chi‐square test (*χ* ^2^ test). Sensitivity, specificity, positive, and negative predictive value of PD‐L2 expression in tissue specimens for diagnosis of malignancy (controls vs patients) are given. Fold change (FC) of PD‐L2 mRNA expression was determined by the ΔΔCT method comparing the average ΔCT values of the two groups.

**Figure 1:**
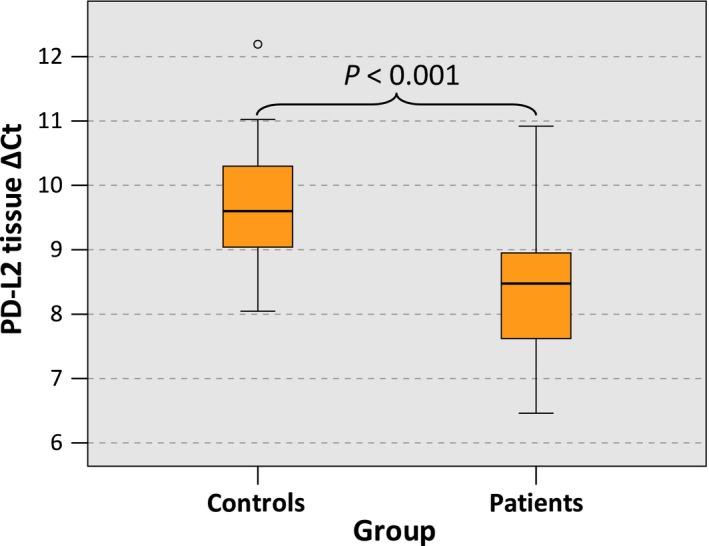
PD‐L2 tissue expression in OSCC patients and healthy mucosal controls. Box plots of the median PD‐L2 expression rates in tumor tissue of OSCC patients (group patients) and healthy oral mucosa of volunteers (group controls). The median ΔCT values of PD‐L2 expression derived from RT‐qPCR are given. Higher ΔCT values indicate lower PD‐L2 mRNA expression. The median, the interquartile range, and the standard deviation are provided. Statistical analyses were carried out by the Mann‐Whitney U test

In OSCC specimens, PD‐L2 expression was significantly higher than in normal oral mucosa (mean ∆CT patients: 7.76, SD 1.33, interquartile range (IQR) 1.34; mean ∆CT controls: 9.16, SD 0.93, IQR 1.27; *P* < 0.001) (Table 3, Figure 1) and a significant 2.65‐fold upregulation of PD‐L2 expression in OSCC was observed (Table 3).

**Figure 2:**
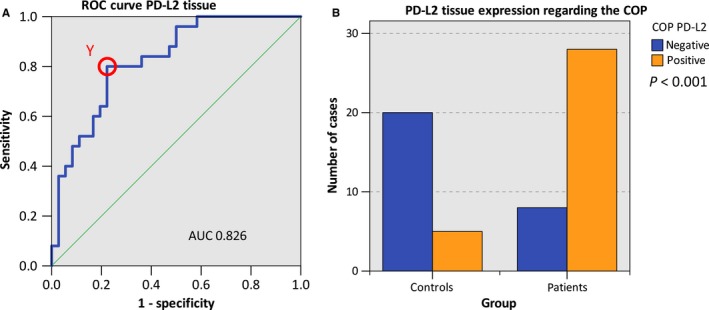
Determination of a cutoff point and allocation of individual cases to a group (controls vs patients) based on PD‐L2 expression. A, ROC curves for PD‐L2 mRNA expression based on the RT‐qPCR data. The diagrams are a plot of the sensitivity (true‐positive rate) vs 1‐specificity (false‐positive rate) over all possible ∆CT values. The circle shows the points of the highest Youden (Y) indices which are associated with the COP (patients vs controls). The AUC value is indicated. ROC: receiver operating characteristic, COP: cutoff point, AUC: area under the curve. B, Division of the test and control group (group patients and group controls) into positive and negative subgroups based on the ascertained COPs of PD‐L2 expressed as ∆CT values. Using the chi‐square test, the specimens were positively (malignant) judged if the values lied below the COP. Increased PD‐L2 expression levels in the tissue of OSCC patients (group patients) compared healthy oral mucosa of volunteers (group controls) were significant

The statistical relevance was confirmed by the AUC value determined by generating a ROC curve (Figure 2A) that mounted to 0.826 (Table 3, Figure 2A). Hence, this analysis confirmed that PD‐L2 expression was of significant diagnostic value for discrimination between healthy volunteers and OSCC patients.

The highest Youden index was 0.578 (Figure 2A). The optimal threshold value (COP) stated as ∆CT standards for distinguishing the patients from the healthy controls was 8.50 (Table 3). A ∆CT value lower than the COP (upregulated PD‐L2 expression) was considered to be positive for malignancy. Based on this COP, the two groups (patients and controls) were separated into positive and negative specimens in order to investigate whether PD‐L2 expression allows the detection of malignancy in a certain sample. Out of the OSCC patients, 77.8% (28/36) showed increased PD‐L2 expression. In contrast, only 20.0% (5/25) of the control samples (normal mucosa) showed increased PD‐L2 expression. The statistical evaluation by the chi‐square test revealed that increased expression rates of PD‐L2 were statistically relevantly associated with malignancy (*P* < 0.001). The results are summarized in Table 3 and illustrated in Figure 2B.

Therefore, increased expression of PD‐L2 in tissue specimens may indicate the existence of OSCC. Data for sensitivity, specificity, positive, and negative predictive values of PD‐L2 expression for diagnosis of malignancy are given in Table 3.

In contrast to the described results in tissue specimens, there was no significant difference in PD‐L2 expression with regard to blood samples of OSCC patients and healthy controls (mean ∆CT patients: 10.83, mean ∆CT controls: 10.95; *P* = 0.797; Table 3).

### 3.3 Association of PD‐L2 expression in tissue and blood samples with histomorphological parameters (T‐, N‐, L‐, Pn‐status, grading) of OSCC patients

Comparison of PD‐L2 expression in OSCC tissue specimens and peripheral blood of OSCC patients with histopathologic parameters (T‐, N‐, L‐, Pn‐status, grading) was carried out.

PD‐L2 expression in tissue specimens showed no difference depending on the N‐status. Additionally, there was no significant association of tissue and blood expression of PD‐L2 and tumor size (T1/T2 vs T3/T4) (*P* > 0.05).

Moreover, PD‐L2 expression in blood samples of OSCC patients suffering from lymph node metastases (N+) did not show higher expression than in blood samples of patients without metastases (N0) (mean ∆CT N+ 10.51, mean ∆CT N0 11.13; *P* = 0.141) (Table 4). Furthermore, no significant changes in blood expression levels of PD‐L2 and histologically proven lymph vessel infiltration (L‐status), perineural infiltration (Pn‐status), and tumor grading (G1‐G3) were observed. The results of statistical analyses are summarized in Table 4.

**Table 4:** PD‐L2 expression in peripheral blood of OSCC patients related to histomorphological parameters (T‐, N‐, L‐, Pn‐status, grading)

	n	Mean	SD	*P*‐value
T‐status				
PD‐L2 tissue	35			0.805
T1‐T2	20	7.65	1.35	
T3‐T4	15	7.80	1.33	
PD‐L2 blood	47			0.431
T1‐T2	29	10.94	1.34	
T3‐T4	18	10.68	1.23	
N‐status				
PD‐L2 tissue	35			0.960
N0	21	7.78	1.03	
N+	14	7.62	1.72	
PD‐L2 blood	47			0.141
N0	25	11.13	1.15	
N+	22	10.51	1.39	
L‐status				
PD‐L2 tissue	35			0.252
L0	27	7.58	1.28	
L1	8	8.20	1.45	
PD‐L2 blood	47			0.692
L0	37	10.77	1.30	
L1	10	11.10	1.29	
Pn‐status				
PD‐L2 tissue	35			0.856
Pn0	20	7.63	1.28	
Pn1	15	7.84	1.42	
PD‐L2 blood	47			0.212
Pn0	29	10.67	1.40	
Pn1	18	11.11	1.07	
Grading				
PD‐L2 tissue	36			0.130
G1	6	8.09	1.11	
G2	19	7.36	1.34	
G3	11	8.26	1.30	
PD‐L2 blood	48			0.828
G1	7	11.29	1.43	
G2	23	10.72	1.36	
G3	18	10.80	1.15	

CT, cycle threshold; grading, histologic tumor grading: G1‐G3; L0, no lymph vessel invasion; L1, lymph vessel invasion present; L‐status, lymph vessel invasion; n, number of cases; N+, lymph node metastases present; N0, no lymph node metastases; N‐status, lymph node metastases; Pn0, no perineural invasion; Pn1, perineural invasion present; Pn‐status, perineural invasion; SD, standard deviation.

Association of PD‐L2 mRNA expression rates in tissue specimens (OSCC tumor tissue) and peripheral blood samples of OSCC patients with histomorphologic parameters of tumor progression (T‐, N‐, L‐, Pn‐status, grading). The mean ΔCT value (mean), standard deviation (SD), and the *P*‐value provided by the Mann‐Whitney U test are shown. Higher ΔCT values indicate lower PD‐L2 mRNA expression.

### 3.4 Kaplan‐Meier analysis of survival

**Figure 3:**
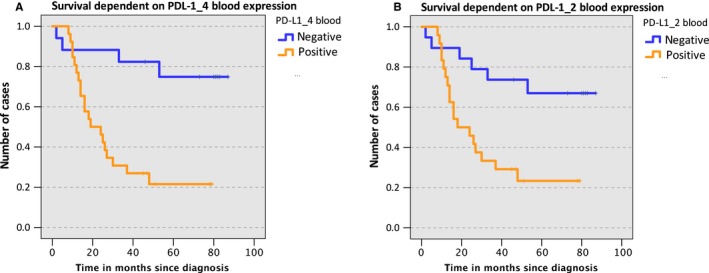
Kaplan‐Meier survival analysis based on PD‐L1 mRNA expression in peripheral blood. A and B, A Kaplan‐Meier survival analysis based on the PD‐L1 mRNA expression in peripheral blood samples above (positive) or below (negative) the COP. Data for PD‐L1 variant PD‐L1_4 (Figure 3A) and variant PD‐L1_2 (Figure 3B) are provided. The COP was determined using the highest Youden (Y) index associated with survival within 3 y. The initial number of eligible cases was 43 for PD‐L1_4 (17 negative and 26 positive) and 43 for PD‐L1_2 (19 negative and 24 positive). OSCC cases with increased PD‐L1 mRNA expression in the peripheral blood (positive) showed inferior survival

No significant association of PD‐L2 expression in tissue and overall survival could be shown (*P* = 0.4). Since expression rates in blood did not correlate with clinical and histomorphological parameters, no Kaplan‐Meier analysis was done using expression values in blood. In contrast to PD‐L2, we could already show that the overexpression of PD‐L1 in peripheral blood samples was significantly associated with the lymph node status.^7^ Additionally, based on our previously reported PD‐L1 expression data in peripheral blood samples of OSCC patients^7^ a significant correlation of PD‐L1 overexpression and 3‐year survival rate was ascertained (*P*_PD‐L1_4_ = 0.03; *P*_PD‐L1_2_ = 0.01). Based on the calculated COP that allows the discrimination of patients with regard to survival, the samples were assessed as positive or negative for the expression of PD‐L1 (*P*_PD‐L1_4_ = 0.01; *P*_PD‐L1_2_ = 0.006). Subsequently, in order to check the influence of increased expression of PD‐L1 in blood on overall survival, a Kaplan‐Meier survival analysis based on the previously reported PD‐L1 expression data in peripheral blood samples of OSCC patients was performed. Survival data analysis depending on expression of the PD‐L1 variants PD‐L1_4 (Figure 3A) and PD‐L1_2 (Figure 3B) was performed applying ΔCT values corresponding to the threshold that indicates survival within 3 years. OSCC cases with increased PD‐L1 mRNA expression in peripheral blood samples (positive) showed inferior survival (*P*_PD‐L1_4_ = 0.01; *P*_PD‐L1_2_ = 0.04). However, a significant association of PD‐L1 expression in tissue with patient survival could not be determined (*P*_PD‐L1_4_ = 0.59; *P*_PD‐L1_2_ = 0.78).

## 4 DISCUSSION

### 4.1 Is there a prognostic significance of PD‐L2 or PD‐L1 expression?

The current study revealed no association between systemic PD‐L2 expression and disease prognosis. However, we could demonstrate an association of 3‐year survival and systemic PD‐L1 expression. PD‐L1 and PD‐L2 expression in peripheral blood samples originates primarily from circulating immune cells. The role of PD‐L1 and PD‐L2 signaling seems to be more complex than just to be two interchangeable ligands that activate PD1 and thus lead to T‐cell inactivation.^22^

Recent findings suggest a novel role for PD‐L2 in enhancing T‐cell immunity.^22^ Blocking PD‐L2 in a malaria mouse model led to increased numbers of regulatory T cells and diminished T‐cell activation.^22^, ^23^ Additionally, PD‐L2 inhibition was associated with increased lethality of the disease.^22^ This underlines our incomplete understanding of the regulation of PD/PD‐L signaling and implies that PD‐L2 might also act as immune activation ligand in certain conditions.

In contrast, no immune stimulatory capabilities of PD‐L1 have been described. Besides PD1, PD‐L1 can also bind to the costimulatory CD80 receptor (B7‐1),^9^, ^24^ while PD‐L2 does not.^25^ CD80 is expressed by antigen‐presenting cells and stimulates the CD28 receptor on T cells that is essential for T‐cell activation.^26^ The interaction of PD‐L1 with CD80 is far less understood than PD‐L1/PD1 signaling.^27^ PD‐L1 expressed on T cells might compete with the CD28 receptor for the costimulatory signal provided by CD80 expressing antigen‐presenting cells.^24^ Binding affinity of CD80 to PD‐L1 is higher than to CD28.^24^ Therefore, PD‐L1 expression of T cells might prevent the CD80‐derived activation of CD28^24^ and thus inhibit T‐cell activation. Specific blocking of PD‐L1/CD80 interaction was shown to enhance T‐cell expansion and activation.^28^ Thus, interaction of PD‐L1 with CD80 could be an important T‐cell inhibiting pathway independent of the PD1 receptor.^25^ This might explain why some in vivo studies discovered a higher effect of anti‐PD‐L1 therapy compared with anti‐PD‐1 or anti‐PD‐L2 antibodies.^9^

The expression of PD‐L1 and PD‐L2 also seems to be regulated differentially. PD‐L1 expression was found to be upregulated by IL‐10 signaling, while this cytokine did not influence PD‐L2 expression.^29^ A recent mRNA expression analysis in peripheral blood leukocytes of patients with multiple sclerosis revealed significantly decreased PD‐L1 expression compared to healthy controls.^30^ However, there was no difference in PD‐L2 expression observable.^30^ This indicates that PD‐L1 might be more relevant for mediating peripheral immune tolerance than PD‐L2. These results also emphasize the possible distinctive roles of PD‐L1 and PD‐L2 in different pathologies.

In conclusion, PD‐L1 seems to have additional immune inhibitory capabilities independent of PD1, while PD‐L2 might act immune activating under certain conditions. This could explain the association of PD‐L1 with survival and the previously described correlation with parameters of malignancy (N‐status, tumor grading),^7^ while there was no significant association of PD‐L2 expression with histomorphologic parameters and survival. Our data indicate that systemic PD‐L1 expression might be more relevant for oral cancer progression than systemic PD‐L2 expression. Therefore, PD‐L1 might be a possible candidate for immune monitoring in OSCC patients. However, there remain open questions that should be addressed in future prospective studies: (a) what is the significance of checkpoint expression in tumor tissue, lymph nodes, or peripheral blood? (b) Is an altered immune checkpoint expression an independent prognostic parameter? (c) Could systemic immune checkpoint expression be used as monitoring parameters to detect recurrences?

### 4.2 Local upregulation of PD‐L2 expression in cancer

PD‐L2 expression is far less studied in human cancer than PD‐L1.^8^ Interaction of PD‐L2 with PD1 expressing T cells leads to an inhibition of T‐cell activation, similar to PD‐L1.^6^, ^8^ A recent immunohistochemical study could detect PD‐L2 expression in a variety of cancer types such as gastric cancer, lung cancer, and HNSCC.^8^ PD‐L2 expression was even present in some cases with missing PD‐L1 expression.^8^ In other malignancies such as primary mediastinal large B‐cell lymphoma and colorectal cancer, an increase in PD‐L2 expression^31^, ^32^ and a negative prognostic value^32^ was also shown. This outlines the potential relevance of PD‐L2 expression as inhibitory immune checkpoint.

The current study reveals for the first time a significant upregulation of PD‐L2 expression in OSCC tissue compared to healthy oral mucosa. Additionally, a significant correlation between PD‐L2 overexpression and malignancy was assigned if each individual tissue sample was determined to be negative (below COP) or positive (above COP) for PD‐L2 expression using the calculated cutoff point. Thus, PD‐L2 expression analysis could be used as diagnostic test for the existence of OSCC in a certain tissue sample. Tumor cells, antigen‐presenting cells as well as some T cells might be the source of PD‐L2 expression in the analyzed tissue specimens.^33^ PD‐L2 expression on T cells seems to be differentially regulated depending on T‐cell polarization. It was shown that PD‐L2 expression can be found on Th2 polarized cells in contrast to Th1, Th17, or T_reg_ cells, which are negative for PD‐L2 expression.^34^ Therefore, the increase in PD‐L2 expression in OSCC could be associated with an increased Th2 polarization of the tumor‐associated T cells. This is consistent with previous reports describing a dominance of Th2 polarization in OSCC.^35^

In a previous study, we could analyze the expression of PD‐L1 in tissue samples and blood of OSCC patients compared to controls.^7^ We could show that PD‐L1 expression was significantly increased in OSCC tissue compared to healthy oral mucosa. However, there was no significant difference in peripheral blood PD‐L1 expression between OSCC patients and controls.^7^ However, PD‐L1 expression in blood samples of OSCC patients correlated significantly with the existence of lymph node metastases (N+).^7^ The current study shows that PD‐L2 is also upregulated in OSCC tissue compared to healthy oral mucosa. Both PD1 ligands—PD‐L1 and PD‐L2—could synergistically contribute to the immunosuppressive local tumor microenvironment in OSCC. In contrast to the upregulation of PD‐L2 in tissue samples, blood samples of OSCC patients and healthy control persons showed no difference in PD‐L2 expression. This is comparable to previous data showing no differential regulation of PD‐L1 in peripheral blood of OSCC patients and controls.^7^

### 4.3 Association between systemic immune dysfunction and disease progression

Immune checkpoint expression can be interpreted as an expression of immunity vs immune tolerance. In this regard, immunity is associated with low inhibitory checkpoint signaling while high checkpoint signaling is characteristic of immune tolerance. In the autoimmune disease, rheumatoid arthritis, PD1 signaling was recently shown to be downregulated.^36^ A therapeutic activation of immune checkpoints is therefore seen as a promising therapeutic option in autoimmune diseases.^36^ In cancer patients, checkpoint inhibition has been one of the most game‐changing therapeutic approaches introduced into clinical care in the last decades.^37^ However, currently checkpoint inhibitors are applied primarily in advanced stage cancer. It is known that advanced tumor stage is associated with increasing immune dysfunction.^38^ Therefore, one could hypothesize that immune checkpoint therapies might be more efficient in early‐stage cancer with a milder immune dysregulation. In patients with bladder cancer, PD‐L1 blocking immunotherapy showed a response rate of 15% if applied to patients that showed progression on platinum‐based chemotherapy. However, patients naïve to cisplatin showed response rates of 24%.^27^ Hence, early‐stage OSCC patients might also profit from a better response rate to checkpoint inhibitors. First studies with neoadjuvant application of the PD1 inhibitor pembrolizumab in surgical resectable HNSCC indicate that response rates might be superior compared to the current second line use in cases refractory to platinum‐based systemic therapies and with exhausted surgical and radiation‐oncological treatment approaches.^39^, ^40^ As pembrolizumab inhibits PD‐L1 and PD‐L2 signaling, this might also be a promising drug for neoadjuvant OSCC treatment.

We could show that high PD‐L1 expression in the peripheral blood of OSCC patients is associated with inferior survival and parameters of increased tumor progression.^7^ However, OSCC patients and healthy control persons showed no difference in PD‐L1 expression in peripheral blood.^7^ Therefore, the degree of PD‐L1 expression could be associated with a status of systemic immune tolerance that is preexisting and independent of the existence of a malignancy. The development of the tumor is triggered by local toxins leading to dysregulation of cellular functions in oral epithelium. However, the progression of the disease might than be determined by a preexisting systemic immunologic status of awareness or immune dysfunction that might be associated with systemic PD‐L1 expression. Currently, there are no established markers available for monitoring immune dysfunction in cancer patients.^38^ We propose that in contrast to PD‐L2, systemic PD‐L1 expression should be investigated as potential parameter for monitoring systemic immune dysfunction in cancer.

The results of the current study outline the effort that needs to be undertaken to better understand the physiologic and pathophysiologic role of PD1 signaling and the therapeutic efforts to establish immune modulating treatment strategies as first‐line therapies in OSCC.

### 4.4 Future perspectives

Future studies should examine the correlation of PD‐L1 and PD‐L2 with the expression of the PD1 receptor. The investigation of further immune regulatory pathways in cancer patients and autoimmune diseases could add to our currently fragmentary knowledge of immune regulation in physiologic and pathologic conditions. Additionally, the value of PD‐L1 as potential immune monitoring parameter for OSCC patients needs to be verified in blood, tissue, and regional lymph nodes in prospective studies. Finally, a possible role of PD‐L1 expression in peripheral blood as predictive parameter for PD1‐blocking immunotherapy in oral cancer should be studied.

## 5 CONCLUSION

Increased expression of the PD1 ligands PD‐L2 in tissue could serve as a diagnostic parameter for the existence of OSCC. The increased expression of PD‐L2 in cancer tissue can be interpreted as an expression of local immune tolerance in the tumor microenvironment. PD‐L2 might act synergistically to PD‐L1 in mediating local immune tolerance.

The current study revealed for the first time, that increased PD‐L1 expression in peripheral blood of OSCC patients is associated with inferior overall survival, while systemic PD‐L2 expression was not associated with histomorphologic and prognostic parameters. Systemic PD‐L1 expression should be further investigated as immune monitoring parameter in OSCC.

A systemic state of immune activation—indicated by low checkpoint expression in the peripheral blood—or a state of immune tolerance—indicated by high checkpoint expression—could determine tumor progression in OSCC. The current study indicates that systemic PD‐L2 expression is a less relevant parameter for OSCC progression than systemic PD‐L1 expression. This could be explained by a different regulation and different binding partners of PD‐L1 and PD‐L2 which are eventually associated with a different tumor‐immunological relevance. The concept of a preexisting systemic immune status determining the prognosis of oral cancer implicates the use of immune modulating therapies in early stages of OSCC.

## CONFLICT OF INTEREST

The authors declare that they have no competing interests.
